# O9.3. PSYCHOTIC EXPERIENCES IN COMMON MENTAL DISORDERS AND AS CLINICAL MARKERS OF RISK FOR SUICIDAL BEHAVIOUR

**DOI:** 10.1093/schbul/sby015.247

**Published:** 2018-04-01

**Authors:** Ian Kelleher

**Affiliations:** Royal College of Surgeons in Irel

## Abstract

**Background:**

A high risk for suicidal behaviour has long been recognized in psychotic disorders. More recently, research has demonstrated that subclinical psychotic experiences are strong markers of risk for suicidal behaviour. Whether PEs are specific risk markers for suicidal behaviour, beyond the indirect risk resulting from co-occurring psychopathology, remains unclear.

**Methods:**

This study used a stratified, multi-stage probability sample of households in England to recruit a nationally representative sample aged 16 years and over (N=7,403). Participants were assessed for psychotic experiences, suicide attempts, common mental disorders and borderline personality disorder/traits.

**Results:**

Psychotic experiences were reported by approximately 4% (n=323) of the total sample and were prevalent across the full range of mental disorders: the highest prevalence in non-psychotic disorders was in individuals with agoraphobia, nearly a quarter of whom reported psychotic experiences. Eighteen percent of individuals with social phobia reported hallucinations, as did 17% of individuals with OCD, 14% of individuals with depression, and 11% of individuals with generalised anxiety disorder. Psychotic experiences were risk markers for suicide attempts, regardless of whether they occurred in individuals with a common mental disorder (OR=2.47, 95%CI=1.37–4.43), individuals without a common mental disorder (OR=3.99, 95%CI=2.47–6.43), individuals with high borderline personality disorder traits (OR=2.23, 95%CI=1.03–4.85) or individuals without significant borderline personality disorder traits (OR=2.47, 95%CI=1.37–4.43).

**Discussion:**

Psychotic experiences are prevalent across a wide range of (non-psychotic) mental disorders. They demonstrate a strong relationship with suicidal behaviour, beyond that explained by co-occurring mental disorder diagnoses.

